# Genetic structure and differentiation in *Dendrocalamus sinicus* (Poaceae: Bambusoideae) populations provide insight into evolutionary history and speciation of woody bamboos

**DOI:** 10.1038/s41598-018-35269-8

**Published:** 2018-11-16

**Authors:** Jun Bo Yang, Yu Ran Dong, Khoon Meng Wong, Zhi Jia Gu, Han Qi Yang, De Zhu Li

**Affiliations:** 1 Research Institute of Resources Insects, Chinese Academy of Forestry, Kunming, Yunnan 650233 China; 2 Germplasm Bank of Wild Species, Key Laboratory of Biodiversity and Biogeography, Kunming Institute of Botany, Chinese Academy of Sciences, Kunming, Yunnan 650204 China; 3 Singapore Botanic Gardens, 1 Cluny Road, Singapore, 259569 Republic of Singapore

## Abstract

Evolutionary processes, speciation in woody bamboos are presently little understood. Here we used *Dendrocalamus sinicus* Chia & J.L. Sun as a model species to investigate dispersal or vicariance speciation in woody bamboos. Variation in three chloroplast DNA (cpDNA) fragments and eight simple sequence repeat markers (SSR) among 232 individuals sampled from 18 populations across the known geographic range of *D. sinicus* was surveyed. *D. sinicus* populations exhibited a high level of genetic differentiation which divided them into two groups that are consistent with different culm types. Eleven haplotypes and two lineages (Straight-culm and Sinuous-culm lineages) were identified from phylogenetic analyses, and a strong phylogeographic structure across the distribution range was found. The demographic and spatial expansion times of the Straight-culm lineage were calculated as 11.3 Kya and 20.8 Kya, respectively. The populations of *D. sinicus* had experienced dispersal and long-term isolation, although this trace was diluted by contemporary gene flow revealed by SSR data. Our results provide an phylogeographic insight to better understand the speciation processes of woody bamboos.

## Introduction

The biggest grasses in the world, bamboos belong to the subfamily Bambusoideae of the Poaceae, and include some 115 genera with more than 1400 species^1,2^. These taxa are naturally distributed in all continents except Europe and Antarctica, and their centers of species diversity are the tropical and subtropical regions of Asia, Africa and South America^2^. Bamboos are a significant natural resource throughout much of the world, providing food and raw materials for construction, paper pulp and manufacturing, etc.^3^. Many woody bamboos have a life cycle peculiar among angiosperms, in having a very long vegetative period of several decades to even 150 years, followed by gregarious flowering and monocarpy^4^. So far, due to lack of fossil material, long vegetative growth periods and unpredictable flowering episodes, the evolution and speciation of woody bamboos have always been an interesting but stubbornly difficult topic^4–7^.

Speciation is crucial to biodiversity and can be categorized in non-geographic and geographic modes^8^. On the aspect of non-geographic mode of bamboos, chromosome doubling and hybridization are generally deemed as the main mechanism for modern species diversity of bamboos^9–11^. In regard to woody bamboos, the chromosome allopolyploid may account for the origin of major lineages and subsequent radiation speciation^10^, for example, the diversity of modern temperate and tropical woody bamboos^10,11^. Meanwhile, the hybrid speciation among woody bamboos has been observed occasionally in the nature^12^. On the other hand, vicariance and dispersal speciation are viewed as two main modes of geographic speciation in plant and animal^13,14^. Based on the modern distribution pattern in the world, the species diversity of bamboos is generally regarded as the result of vicariance speciation^7,14^, which was supported by evidence of molecular bamboo systematics^4,10,11^. However, does dispersal speciation happen at the level of species among woody bamboos? As far as we know, there is little research report in this field. To further understand this question, more comprehensive studies are required, especially at the population level.

In this study, we use *Dendrocalamus sinicus* Chia & J. L. Sun as a model to gain further insights into this topic. *D. sinicus* is among the largest bamboos known in the world, with culms reaching over 30 m high and 30 cm in diameter, and has been acknowledged as the strongest bamboo known^15^. This typical paleotropical woody bamboo occurs naturally only at elevations of 600–1,500 m in south and southwestern Yunnan Province in southwest China^16^. *D. sinicus* also seems to have an interesting variation from its more northerly distribution to the south based on culm shape. The more northerly populations in southwestern Yunnan have the typically straight culms of erect clumped bamboos, whereas in the more southerly populations, clumps often develop culms that are not straight, with the lower half frequently including curved portions giving somewhat sinuous internode sequences^16^ (Fig. S1). Additionally, in intermediate areas between the main localities of these two variants, *D. sinicus* clumps have been observed to bear a mixture of both straight as well as sinuous culms^17^.

How did this variation and its distribution come about? Although both dispersal and vicariance have been suggested as significant in shaping the evolution and biogeography of bamboos^7,14^, there is as yet very little research into speciation and evolutionary processes for this important group of plants. The recent demonstration of molecular techniques in elucidating the phylogenetics, taxonomy and evolutionary history of bamboos and other plant species^18–23^ has prompted our own inquiry, and the relatively limited distribution of *D. sinicus* makes it of special interest for studying the pattern of differentiation in the world’s biggest woody bamboo. The use of maternally inherited chloroplast DNA (cpDNA) in phylogeographic analyses can help infer historical range shifts and recolonization routes of a species^18^. Biparentally inherited nuclear simple sequence repeat variations (SSR) are more powerful for understanding ongoing demographic processes such as contemporary gene exchange^24^.

In the present paper we assemble molecular methods (using cpDNA and SSR markers) and migration patterns between populations in order to reveal the contemporary population structure of *D. sinicus*, to infer its evolutionary history, and to assess the main genetic characteristics that could help inform woody bamboo geographic speciation, i.e. dispersal or vicariance speciation.

## Results

### cpDNA data

**Figure 1:**
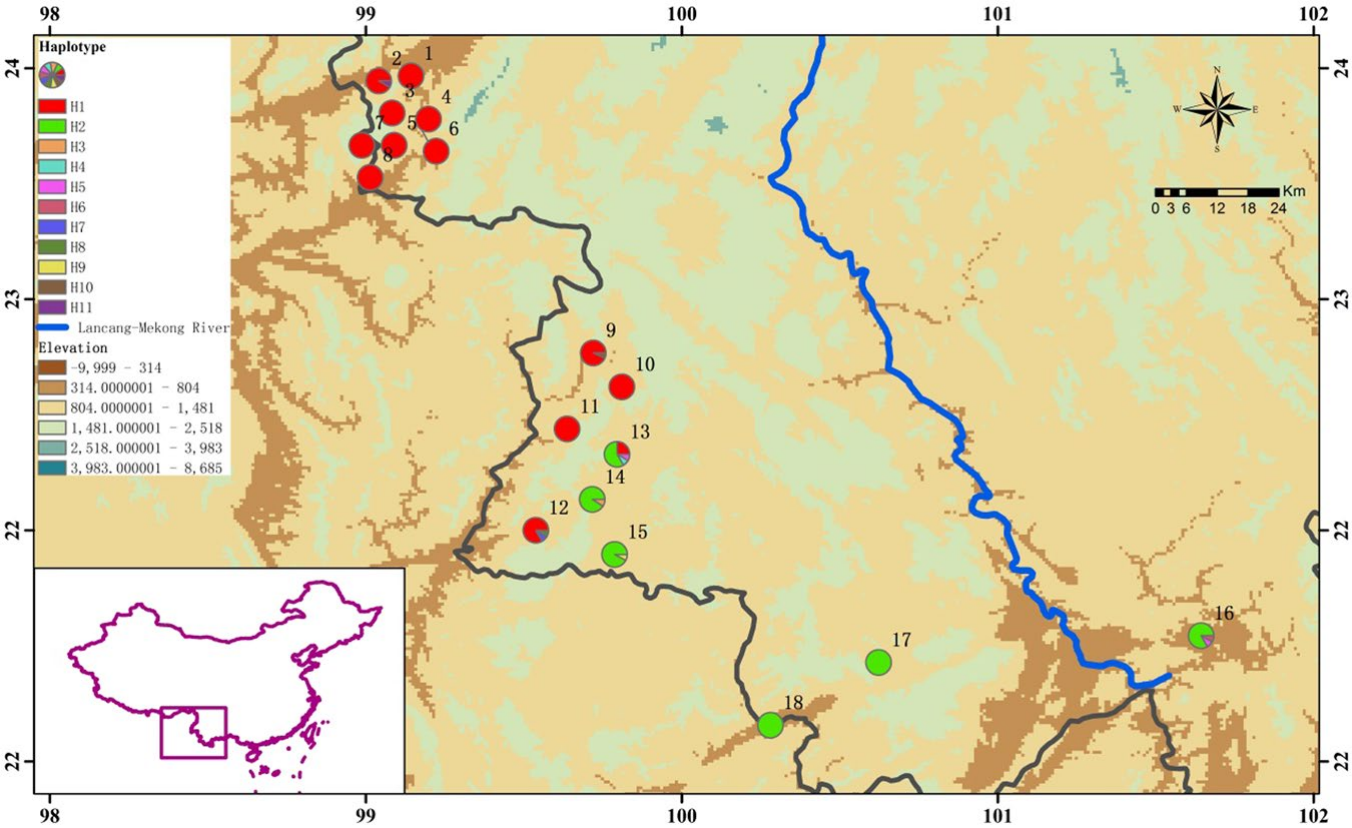
Haplotype distribution in 18 *Dendrocalamus sinicus* populations based on combined sequences of three loci. Colored circles represent the population locations. Maps were drawn using the software ArcGIS version 10.2 (http://desktop.arcgis.com) and modified using Photoshop (Adobe Corporation, California, America).

A total of 1947 bp combined cpDNA sequences were aligned which resulted in 10 polymorphic sites and 11 haplotypes (Tables S1 and S2). The geographic distribution of haplotypes among 18 *Dendrocalamus sinicus* populations is shown in Fig. 1. The most frequent haplotype H1 was found in 158 individuals (68.1% of all samples), mainly among the northern populations. Another common haplotype was H2 which occurred in 64 individuals (27.6% of all samples) from southern populations. H5 was shared by populations 13 and 16. Haplotypes 3, 4, 6, 7 and 8, 9, 10, 11 were exclusive to populations 14, 13, 16, 12, 12, 15, 9, 2, respectively.

**Figure 2:**
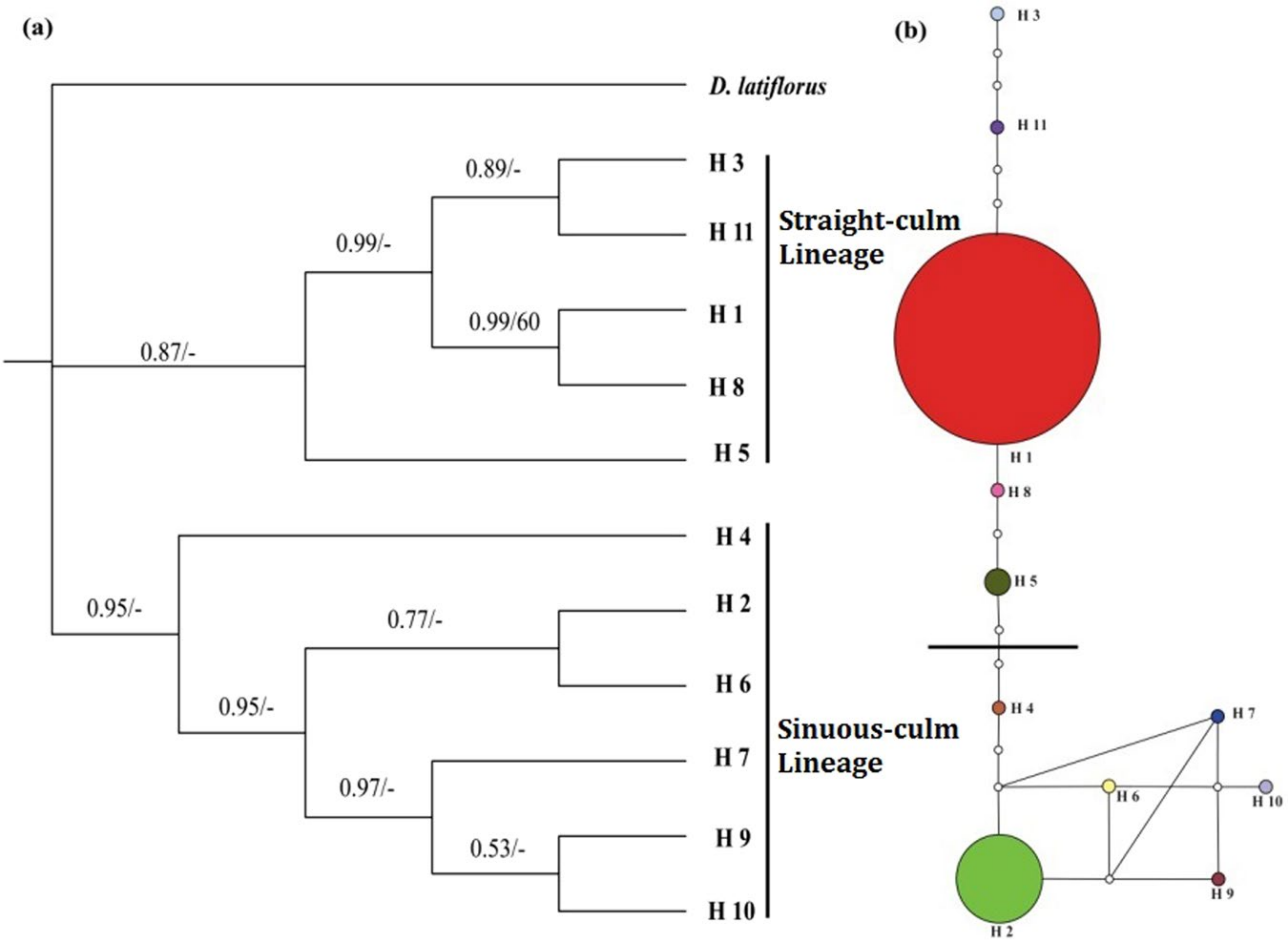
(**a**) Strict consensus tree obtained by analysis of 11 cpDNA haplotypes of *Dendrocalamus sinicus*, with *D. latiflorus* used as outgroup. The numbers on the left of the dash sign (/) indicate posterior probabilities from the Bayesian analysis; the numbers on the right indicate bootstrap values (>50%). (**b**) The 95% confidence network of 11 cpDNA haplotypes. The size of circles corresponds to the frequency of each haplotype. Each solid line represents one mutational step that interconnects two haplotypes for which parsimony is supported at the 95% level. The small open circles indicate hypothetical missing haplotypes.

Maximum parsimony analysis of the 11 cpDNA haplotypes of *D. sinicus* and the outgroup *D. latiflorus* resulted in three most parsimonious trees, each with a length of 26 steps, a consistency index (CI) of 0.500, and a retention index (RI) of 0.683. The Bayesian (BI) tree agreed with the MP tree at nearly all nodes. The topology of the strict consensus tree (Fig. 2a) indicates a single deep phylogenetic split between haplotypes of *D. sinicus*. Although the clades were not well supported (bootstrap values < 50%), they did show high posterior probability values. Haplotypes H2, 4, 6, 7, 9, and 10 formed a clade with 0.95 posterior probability; the other five haplotypes formed another clade with 0.87 posterior probability. Five populations (9, 12, 13, 14 and 16) have haplotypes from both lineages, and all of them were distributed in the central region except for 16 (southern region). The network analysis (Fig. 2b) revealed a similar structure to the phylogenetic tree (Fig. 2a) and segregated all haplotypes into two lineages, here called the Straight-culm Lineage and Sinuous-culm Lineage. Based on their interior location and high frequencies^25^, H1 and H2 appeared to be the ancestral types for the two lineages. Within each lineage, H8 and H11 were derived from H1; and H4, H6, H7 and H9 were derived from H2.

The result of the SAMOVA analysis indicated that two groups of populations (K = 2) were the best grouping option with the highest F_CT_ value (Fig. S2). One group (named the TZ group, population components see Table 1) was assigned the 12 H1-dominant populations with straight culms; another group (named the WQ group) was assigned the remaining six H2-dominant sinuous-culmed populations. AMOVA indicated that most of the variation (93.13%) related to differences between the two groups, 0.88% among populations within groups and 5.99% within populations (Table 2).

**Table 1:** Location of the *Dendrocalamus sinicus* populations studied in Yunnan Province of China, their haplotype profiles and culm type identified based on morphological characters and molecular markers.

Group/population code	Locations	n	Haplotypes (number of individuals)	N_P_	Altitude/m	Latitude (N)	Longitude (E)	culm type identified	
								By morphological characters	By molecular markers
**TZ group**									
1	Mengding, Genma	12	H1 (12)	0	545	23°30′25″	99°01′14″	All straight	All straight
2	Zhupengzhai, Cangyuan	12	H1 (11), **H11** (1)	3	1248	23°26′54″	98°56′17″	All straight	All straight
3	Nanla, Cangyuan	12	H1 (12)	0	1475	23°22′27″	98°58′31″	All straight	All straight
4	Nanban, Cangyuan	14	H1 (14)	0	1126	23°19′12″	99°04′40″	All straight	All straight
5	Yinpan, Cangyuan	12	H1 (12)	2	1302	23°18′32″	98°58′52″	All straight	All straight
6	Banhong, Cangyuan	15	H1 (15)	0	1165	23°17′42″	99°05′58″	All straight	All straight
7	Shangbanlao, Cangyuan	13	H1 (13)	0	1080	23°15′04″	98°56′03″	All straight	All straight
8	Xiabanlao, Cangyuan	12	H1 (12)	1	1018	23°13′17″	98°54′50″	All straight	All straight
9	Zhongke, Ximeng	15	H1 (14), **H10** (1)	3	1295	22°43′41″	99°32′29″	All straight	All straight
10	Mengsuo, Ximeng	15	H1 (15)	0	1080	22°37′58″	99°37′20″	All straight	All straight
11	Wenggake, Ximeng	15	H1 (15)	0	1265	22°30′51″	99°28′03″	All straight	All straight
12	Mengma, Menglian	12	H1 (10), **H7** (1), **H8** (1)	2	944	22°13′45″	99°22′46″	Mixed, all straight except individual 12-5 sinuous (“12” indicates population number)	Mixed, all straight except individual 12-5 sinuous
**WQ group**									
13	Jingxin, Menglian	12	H1 (3), H2 (7), **H4** (1), H5 (1)	1	1007	22°26′36″	99°36′31″	Mixed, all sinuous except individual 13-2, 13-8, 13-9 and 13-12	Mixed, all sinuous except individual 13-2, 13-8, 13-9 and 13-12
14	Dengzhanzhai, Menglian	11	H2 (10), **H3** (1)	1	1010	22°18′59″	99°32′19″	All sinuous	Mixed, all sinuous except individual 14-3
15	Mangxin, Menglian	13	H2 (12), **H9** (1)	0	999	22°09′40″	99°36′05″	All sinuous	All sinuous
16	Menglun, Mengla	12	H2 (10), H5 (1), **H6** (1)	0	543	21°55′59″	101°15′6″	All sinuous	Mixed, all bending except individual 16-9
17	Menghun, Menghai	16	H2 (16)	1	1503	21°51′28″	100°20′40″	Mixed, all sinuous except individual 17-5, 17-14	All sinuous
18	Daluo, Menghai	9	H2 (9)	1	585	21°40′51″	100°02′24″	All sinuous	All sinuous
*D.Latiflorus*	Kunming, Yunnan, China	1			1952	25°07′59″	102°44′31″		

N_P_, exclusive alleles generated from the SSR data set; bold notations in the haplotypes column represent the exclusive haplotypes detected. “Mixed” in “culm type identified” indicates that populations are comprised of both straight- and sinuous-culmed bamboo clumps. The straight-culmed morphotype included cpDNA haplotypes H1, H3, H5, H8 and H11; and sinuous-culmed morphotype included haplotypes H2, H4, H6, H7, H9 and H10.

**Table 2:** Analysis of Molecular Variance (AMOVA) for cpDNA and SSR data of 18 *Dendrocalamus sinicus* populations. *Significant at P < 0.001.

Data	Source of variation	Degrees of freedom	Sum of squares	Variance component	Percentage of variation (%)	Fixation index (F_ST_)
cpDNA	Among groups	1	375.795	3.74874	93.13	0.940*
	Among populations within groups	16	11.158	0.03541	0.88	
	Within populations	214	51.642	0.24132	5.99	
SSR	Among groups	1	101.485	0.42502	13.29	0.306*
	Among populations within groups	16	264.224	0.55497	17.35	
	Within populations	446	989.311	2.21819	69.36	

The estimates of haplotype, nucleotide diversity (Hd and π) and genetic diversity parameters are given in Tables 3 and 4. Hd ranged from 0.000 to 0.636, and π from 0.000 to 0.221. At the species level, Hd was 0.462, π was 0.00196, H_S_ was 0.106 (±0.047), and H_T_ was estimated as 0.497 (±0.0843). At the group level, the WQ group exhibited higher genetic diversity (0.231, 0.00067, 0.215 and 0.238 for Hd, π, H_S_ and H_T_) compared with the TZ group (0.050, 0.00058, 0.052, 0.077). We also detected highly significantly (P ≤ 0.01) larger N_ST_ values (0.875) compared to G_ST_ (0.787), indicating there is a strong phylogeographic structure across the distribution range of *D. sinicus* (Table 4). At the group level, the WQ group also showed a significantly strong (P ≤ 0.05) phylogeographic structure, but this was not manifest in the TZ group.

**Table 3:** Haplotype and nucleotide diversity, allelic richness and heterozygosity measures for the *Dendrocalamus sinicus* populations studied. Hd, haplotype diversity; π, nucleotide diversity; N_A_, number of alleles; N_E_, number of effective alleles; A_R,_ allele richness; H_O_, observed heterozygosity; H_E_, expected heterozygosity; F_IS_, inbreeding index.

Pop. code	*Cp* DNA data		SSR data					
	Hd	π × 10^−2^	N_A_	N_E_	A_R_	H_O_	H_E_	F_IS_
1	0.000	0.000	25	2.491	3.040	0.510	0.525	0.129
2	0.167	0.026	32	2.541	3.640	0.479	0.549	0.150
3	0.000	0.000	28	2.713	3.390	0.458	0.593	0.296
4	0.000	0.000	27	2.641	3.310	0.375	0.554	0.449
5	0.000	0.000	33	2.802	3.930	0.448	0.588	0.351
6	0.000	0.000	29	2.577	3.470	0.433	0.572	0.317
7	0.000	0.000	28	2.735	3.420	0.519	0.577	0.204
8	0.000	0.000	25	2.435	3.080	0.417	0.473	0.325
9	0.133	0.034	33	2.669	3.630	0.425	0.523	0.420
10	0.000	0.000	30	2.665	3.400	0.567	0.519	0.077
11	0.000	0.000	32	2.294	3.520	0.508	0.499	0.147
12	0.318	0.067	36	2.802	4.100	0.521	0.580	0.126
13	0.636	0.221	37	2.971	4.400	0.539	0.629	0.162
14	0.182	0.047	31	2.666	3.720	0.523	0.596	0.215
15	0.154	0.016	28	2.612	3.420	0.567	0.577	0.121
16	0.318	0.067	36	2.707	4.200	0.530	0.559	0.215
17	0.000	0.000	31	2.228	3.370	0.469	0.471	0.292
18	0.000	0.000	18	1.880	2.250	0.500	0.356	−0.378
Overall	0.462	0.196	78	2.580	3.520	0.488	0.541	0.214
TZ group	0.050	0.058	61	2.606	3.495	0.472	0.545	0.250
WQ group	0.231	0.067	57	2.511	3.562	0.521	0.531	0.137

**Table 4:** Genetic diversity parameters for the *Dendrocalamus sinicus* populations studied.

Group	*Cp* DNA data					SSR data			
	H_S_	H_T_	G_ST_	N_ST_	P (G_ST_ < N_ST_)	H_S_	H_T_	G_ST_	G’_ST_
Total	0.106	0.497	0.787	0.875	0.006**	0.567	0.743	0.237	0.247
TZ group	0.052	0.077	0.015	0.028	0.190 (n.s.)	0.572	0.712	0.197	0.211
WQ group	0.215	0.238	0.098	0.172	0.022*	0.557	0.636	0.124	0.146

H_S_, mean genetic diversity within populations; H_T_, total genetic diversity; G_ST_, genetic differentiation using only allelic frequencies; N_ST_, genetic differentiation considering similarities between the haplotypes; G’_ST_, standardized measure of genetic differentiation. n.s., not significant; *Significant (P ≤ 0.05); **highly significant (P ≤ 0.01).

**Figure 3:**
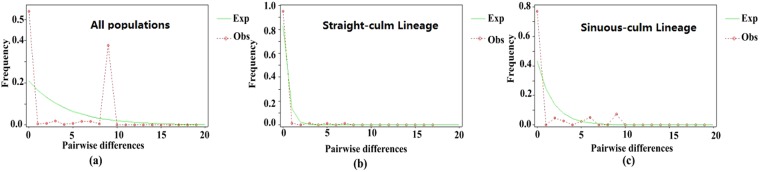
Distribution of the number of pairwise nucleotide differences for cpDNA sequence data in: (**a**) all populations (**b**) the TZ lineage (**c**) the WQ lineage of *Dendrocalamus sinicus*. The green solid line shows observed values, whereas the red dashed line represents expected values under a model of sudden (stepwise) population expansion.

The mismatch distribution of cpDNA haplotypes was clearly bimodal and differed strongly from that predicted by a model of sudden population expansion (Fig. 3a). This prediction was also reflected in positive and non-significant D and Fu’s Fs values (Table 5). These data suggest relatively stable population sizes in *D. sinicus*. At the group level, populations from the WQ group also stayed stable, revealed by a clearly multimodal pattern in the mismatch distribution analysis (Fig. 3c), in accordance with negative and non-significant D and Fu’s Fs values (Table 5). Thus, the WQ group accorded with the assumptions of neutrality. In contrast, the unimodal mismatch distribution for the TZ group was found to be consistent with the expected distribution in the expansion model (Fig. 3b) and coupled with significantly negative values of D and Fs (Table 5), suggesting possible recent population expansion. Based on the value of τ, the demographic and spatial expansion times of the TZ group were calculated as 11.3 Kya and 20.8 Kya, respectively (Table 5).

**Table 5:** Results of the mismatch distribution analysis and neutrality tests of *Dendrocalamus sinicus* at the species and lineage levels.

Pop.	Expansion types	Tau (τ)	Time since expansion began(t, ka)	SSD	P-value	HRI	P-value	D	P-value	Fs	P-value
All pop.	Demographic expansion	—	—	0.20938	0.030	0.565	0.290	2.950	0.977	3.118	0.975
	Spatial expansion	—	—	0.10432	0.180	0.565	0.540				
Straight-culm lineage	Demographic expansion	3.000	11.273	0.00112	0.090	0.881	0.880	−1.951	0.000**	−3.867	0.014*
	Spatial expansion	5.539	20.816	0.00023	0.280	0.881	0.760				
Sinuous-culm lineage	Demographic expansion	—	—	0.03312	0.130	0.609	0.620	−0.979	0.123	−0.629	0.410
	Spatial expansion	—	—	0.00723	0.460	0.609	0.850				

SSD, sum of squared deviation under expansion model; HRI, raggedness; D, Tajima’s D statistic; Fs, Fu’s FS statistic. *Significant (P ≤ 0.05); **highly significant (P ≤ 0.01).

### SSR data

For the eight microsatellite loci used in our research, 68.8% population-locus combinations deviated significantly from HWE.

A total of 78 alleles for the eight loci were detected in 232 samples, among which 14 were exclusive (Table 1). Genetic diversity estimates varied among populations and groups (Table 3). Values for numbers of alleles (N_A_) ranged from 18 to 37, effective numbers of alleles (N_E_) from 1.88 to 2.97, observed heterozygosity (H_O_) from 0.375 to 0.567 and expected heterozygosity (H_E_) from 0.356 to 0.629. Allelic richness (A_R_) estimates among all populations ranged from 2.25 to 4.40. Inbreeding index values ranged from −0.378 to 0.449, which indicate considerable variation in the intensity of inbreeding among populations (Table 3).

At the species level, total genetic diversity H_T_ = 0.743, and mean genetic diversity within populations H_S_ = 0.567 (Table 4). Diversity estimates for the TZ group (H_T_ = 0.712, H_S_ = 0.572) were a little higher than for the WQ group (H_T_ = 0.636, H_S_ = 0.557). Coefficients of gene differentiation (G_ST_ and G’_ST_) were also calculated in Table 4.

**Figure 4:**
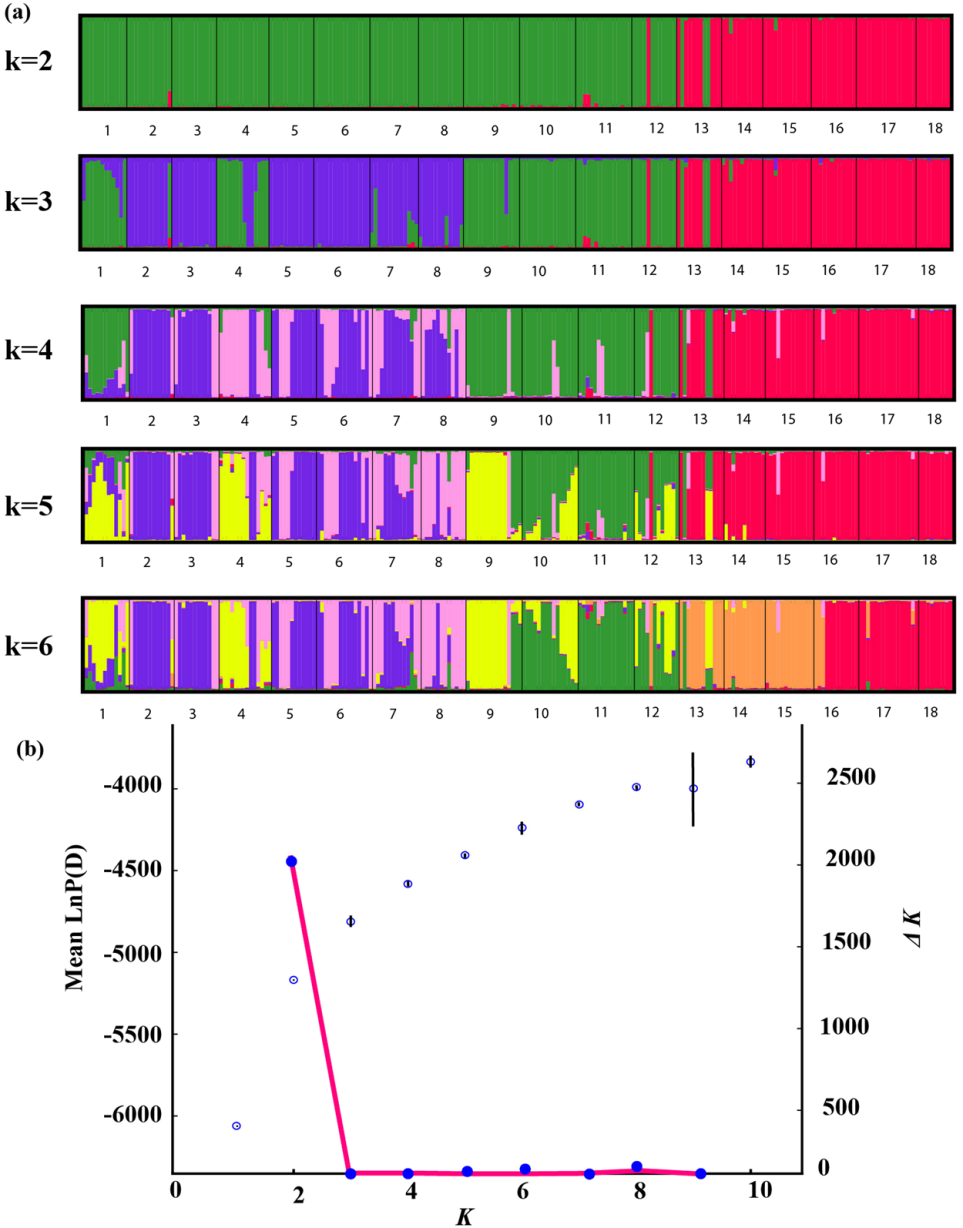
Bayesian clustering results of the STRUCTURE analysis for nSSR data of 18 populations of *Dendrocalamus sinicus* (232 individuals). Number of clusters (K) was from one to 10 in 20 independent runs. (**a**) STRUCTURE-estimated genetic clustering (K = 2, 3, 4, 5, 6), each vertical bar represents an individual and its estimated proportion in K clusters. Black lines separate different populations. Population codes are identified in Table 1. (**b**) Dot plot representing the mean estimate of Ln posterior probability of data for K = 1–10 (20 replicates), the standard deviation of each mean *L(K)* value is given as a black vertical line; the pink line represents distribution of ΔK.

The STRUCTURE analysis showed that Log-likelihood values of the data increased considerably when raising K = 2 (Fig. 4a) and began to flatten out afterwards. Based on the second-order rate of change in values of K (ΔK), the most likely number of genetic clusters for the complete dataset was also estimated at 2. The likelihood Ln (K) averaged over replicates increased with K, and the uptrend reached a plateau after K = 2, suggesting that K = 2 is the probable true number of clusters (Fig. 4b). Although the MedMeaK and MaxMeaK methods suggested K = 5 and 6 to be the best configurations respectively (Fig. S3), our sample collection did not conform to the condition of uneven sample size, and here we have adopted K = 2 to divide clusters.

**Figure 5:**
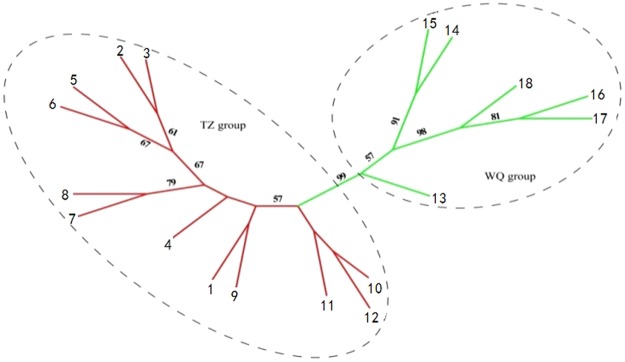
Neighbor-joining tree representing the genetic relationships among 18 populations of *Dendrocalamus sinicus*, based on Nei’s (1987) unbiased genetic distance (D) calculated from SSR data. Population codes are identified in Table 1, and branches are color-coded as in Figs 4 and 5. Numbers by nodes are bootstrap values (>50%) from 1000 replicates.

Meanwhile, the population-based NJ tree (Fig. 5) also suggested a strong pattern of regional differentiation with a high bootstrap value (99.1%) separating the TZ and WQ groups. Moreover, PCO of the SSR phenotypes of *Dendrocalamus sinicus* (Fig. S4) separated all individuals from the TZ and WQ groups along the first axis (PCO1, explaining 28.53% of the total variance), and both groups were separated from the central along PCO2 (18.45%).These confirmed the existence of two distinct genetic units corresponding to the Straight-culm and Sinuous-culm lineages, with the exception of a few individuals (Fig. S4).

The AMOVA results indicated significant genetic differentiation (F_ST_ = 0.306, P < 0.001), with 13.29% of the variation among groups, 17.35% of the variation among populations within groups, and the rest of the variation (69.36%) within populations (Table 2). The results of isolation by distance at the species and regional levels are given in Fig. S5; there is a significant effect of isolation by distance at the species level (R = 0.419, P = 0.003, Fig. S5a) and for the TZ group (R = 0.412, P = 0.007, Fig. S5b). However, this relationship disappeared with respect to the WQ group (R = 0.322, P = 0.273, Fig. S5c).

The BARRIER analysis revealed two possible genetic boundaries; however, the bootstrap support of less than 49.4% (<50%) suggested no significant genetic barriers were detected through the distribution area (Fig. S6).

Bottleneck analysis detected seven population having experienced recent bottleneck effects in both groups (Table S3). Five of them (populations 1, 3, 4, 5, 7) were from the TZ group and the other two (populations 15, 18) were from the WQ group.

### Estimations of migration patterns among three regions: the Northern (main distribution of TZ lineage), Southern (main distribution of WQ lineage) and Intermediate regions

The result of the MIGRATE-N analysis indicated that based on cpDNA data, migration rates (with 95% confidence) between the Northern, or Southern, and the Intermediate populations were largely asymmetrical, with migration from the Intermediate populations into the Northern (m = 0.681) or the Southern populations (m = 0.695) typically greater than in the reverse direction (0.617 and 0.623 respectively, Table S4). The estimated number of migration events per generation was 153064 for cpDNA. On the other hand, based on SSR data, the number of migration events per generation estimated was 1827, and migration from the Intermediate populations into the Northern populations (m = 0.469) was greater than in the reverse direction (m = 0.372). However, migration from the Intermediate (m = 0.396) into the Southern populations was lower than in the reverse direction (Table S4). This suggests that populations of *Dendrocalamus sinicus* can exchange alleles through migration, although it is not to the same extent among different regions. As a whole, migration rates from the Intermediate populations into the Northern or Southern populations were higher than that in the reverse directions.

The migration models test with MIGRATE-N indicated that ancestral gene flow direction was from the Intermediate regions to northern or southern regions based on cpDNA data. However, a full model with two population sizes and two migration rates was supported by SSRs data (Table S5).

Additionally, the pollen-to-seed migration ratio (r = mp/ms) among *D. sinicus* populations was estimated as 9.89.

## Discussion

### Genetic structure and differentiation among *Dendrocalamus sinicus* populations

At the species level, *Dendrocalamus sinicus* shows high total genetic diversity represented by H_T_ = 0.497 (cpDNA) and 0.743 (SSR), and the mean genetic diversity among populations is much higher than that within populations (Table 4). The results of the NJ tree, Bayesian clustering and PCO analysis based on SSR genotyping also indicated that the species has differentiated into two distinct groups: the TZ group (red coded in Figs 4, 5 and S5) and WQ group (green coded). The two markers both revealed the same split into two groups corresponding with distinct culm morphological differences. The total genetic diversity of the WQ group is obviously higher than that of the TZ group based on cpDNA and SSR data (Tables 2 and 4), which is consistent with results of Li *et al*.^26^. It is worth noting an obvious discordance between the two data sets (Tables 2 and 4). For the SSR data, there was 13.29% of variation between the two groups, whereas the extent was 93.13% in cpDNA. This conflict may provide us an insight into the demographic history of *D. sinicus*. Non-recombinant cpDNA allows an inference of historical range shift and recolonization routes^27,28^, unlike SSR markers which can reveal more on contemporary gene flow between populations^20^.

At the group level, a pronounced phylogeographic structure was also uncovered by the genetic differential coefficient within the WQ group and the total populations based on cpDNA data (Table 4), implying a long period of genetic and geographic isolation among *D. sinicus* populations. In addition, the SSR data detected positive isolation by distance among all populations and within the TZ group (Fig. S5), which was in accord with the breeding system and pollination characteristics of this species^29^, i.e., highly restricted pollen transmission and seed dispersal among populations under natural conditions, especially within the WQ group. In nature, the populations of *D. sinicus* only disperse over relatively short distances with limited seed dispersal^16,17^.

On the other hand, the genetic differentiation of *D. sinicus* populations (G_ST_ = 0.787, Table 4) is larger than the average genetic differentiation in angiosperms using maternally inherited markers (G_ST_ = 0.637)^30^, implying lack of seed transmission among *D. sinicus* populations. With biparentally inherited markers, the detected genetic differentiation (G_ST_ = 0.237) of *D. sinicus* was slightly less than *D. membranaceus* (G_ST_ = 0.252)^31^, and far less than *D. giganteus* (G_ST_ = 0.847) and *D. brandisii* (G_ST_ = 0.842)^32^. The different results from plastid and nuclear DNA imply that the pollen transmission may far exceed seed dispersal in the sexual reproduction of *D. sinicus*^17^.

Moreover, the pollen-to-seed migration ratio (r = mp/ms) estimated among populations of *D. sinicus* (r = 9.89) was much less than the average among Angiosperms and Conifers assessed (mean = 17)^30^. The contemporary probability of hybrids between the TZ and WQ groups in the wild would appear to be minimal. During the last c. 30 years, sporadic flowering and seed set in TZ populations of *D. sinicus* have been observed in natural populations in southern Yunnan, whereas sporadic flowering was rare with almost no seed set among WQ populations^17,33^. Our own observations of several flowering clumps of *D. sinicus* in the wild showed that their flowering times were not synchronous, which could explain a lack of pollination and seed set^29^. What is more important is that, based on cpDNA and SSR data, both groups of *D. sinicus* showed significant genetic differentiation which was consistent with geographic isolation.

### Lineage divergence and demographic history

Lineage divergence and demographic history can provide insight into the speciation process^18–20^. The SAMOVA analysis (Fig. S2), tree topology (Fig. 2a) and network analysis (Fig. 2b) of cpDNA data are consistent with the hypothesis that *Dendrocalamus sinicus* was initially split into two lineages, i.e., the Straight-culm and Sinuous-culm lineages. Among 18 extant populations, a total of 11 populations (61.1%) possessed at least one exclusive haplotype or allele (Table 1), indicating a long period of isolation between populations. AMOVA analysis of cpDNA shows 93.13% variation between groups. Meanwhile, there were five mixed populations (with haplotypes from both lineages) based on cpDNA data, with four of them (populations 9, 12, 13 and14) from the intermediate area between the main localities of the lineages, and one (population 16) located in the southernmost area. The results of bidirectional migration rates analyses (Table S4) showed that the main migration directions were from the intermediate area to the north and south parts of its distribution, which indicated that the intermediate area of the two lineages may be the center of origin of *D. sinicus*. Interestingly, populations 13 and 16, separated by 178.4 km in distance and the Lancang-Mekong River, shared a rare chloroplast haplotype H5 which clustered in the Straight-culm lineage (Table 1, Fig. 2a). Seed exchange between these two populations under natural conditions seems impossible, so a possible explanation may be either human-mediated introduction which allowed secondary contact between the two lineages^16^.

The records of the Guliya ice core indicated that the Younger Dryas cooling event affecting most areas of the Northern Hemisphere ended around 10.9 Kya ~ 10.8 Kya BP, marking the end of the last glacial period and passage into the Holocene^34^. The mismatch distribution test and neutrality test of *D. sinicus* populations both detected expansion within the Straight-culm lineage (Fig. 3, Table 5), with an estimated time around 11.3 Ka (under the demographic expansion model), which on the whole kept the same step with the end of the Younger Dryas cooling event. It may be a result that the Straight-culm lineage which occurred in cool areas can adapt to the warmer area in the south, while the Sinuous-culm lineage may not be able to survive with a relatively cooler climate at higher latitudes in the northern area of its distribution^16^.

### Evolutionary and speciation implications

In the present study, a strong phylogeographic structure across the distribution range of *Dendrocalamus sinicus*, with the Straight-culm and Sinuous-culm lineages, has been confirmed through phylogenetic analyses of cpDNA and SSR variation. By and large, the distribution areas of this species are divided into three regions, namely the northern, the southern and the intermediate regions (Fig. 1). The migration model analyses using MIGRATE-N indicated that ancestral gene flow direction was mainly from the Intermediate regions to northern or southern regions based on CpDNA data (Tables S4 and S5). Meanwhile, almost all private haplotypes occurred at the Intermediate regions (Table 1, Fig. 2), and the genetic diversity within the northern (main part of TZ group) and southern populations (main part of WQ group) were much lower than that of the populations from the Intermediate regions (Tables 3 and 4), suggesting that the Intermediate region is a diversity center of this species and a founder effect existing within the TZ group.

Reviewing the geological and biogeological events associated with the distribution area of *D. sinicus*, we found that the geological conditions of this area have been stable since the late Tertiary^35–37^, and there is no large river and mountain barrier between the distribution areas^38^. Furthermore, no strong genetic barrier was detected using SSR data (Fig. S5) in this study, which indicated no obvious geographical condition for vicariance speciation in *D. sinicus*. Recent phylogenetic study of the flora of southern Yunnan also illustrated that geological changes and associated dispersals across biomes have existed since the late Tertiary^37^. Meanwhile, hybridization between two culm-shape lineages has not been observed in different variants in the past thirty years^16,17,33^.

To sum up, the relatively independent distribution areas of different culm-shape types of *D. sinicus* shows that this bamboo species is experiencing a process of dispersal speciation, which provides a good case for studying speciation among woody bamboos. Therefore, these results imply that the overall extant genetic diversification of *D. sinicus* could be due to long distance dispersal, rather than vicariance. The modern distribution pattern of two culm types is probably the result of different altitude and latitude conditions associated with populations of two lineages, respectively (Table 1).

## Materials and Methods

### Plant material sampling

We collected leaf samples from 232 individuals (clumps) of 18 populations covering the entire geographic range of *Dendrocalamus sinicus* (Fig. 1, Table 1). Nine to 16 individuals/clumps at least 100 m apart from each other were sampled in each population. Employing the concept of McClure^39^, we treated each clump as a potential genet and the culms within as ramets of a clone, and we assumed that an assortment of genetic material existed within each population. Fully developed leaves collected for DNA extraction were rapidly dried and preserved in silica gel. Vouchers for each population were deposited at the Herbarium of the Research Institute of Resources Insects, Chinese Academy of Forestry. *Dendrocalamus latiflorus* was chosen as outgroup in the cpDNA haplotypes analysis, based on previous phylogenetic study^40^.

### Laboratory procedures

We extracted genomic DNA using a modified 4x cetyltrimethyl ammonium bromide (CTAB) extraction protocol^41^. Three cpDNA intergenic spacer regions with rich information, i.e., *rpl*32-*trn*L, *rbc*L-*psa*I and *trn*G-*trn*T, were chosen for sequencing based on Zhang *et al*.^42^. The 20 µl PCR mix contained 10 µl 2× PCR buffer including KCl_2_ (100 mM), MgCl_2_ (3 mM), dNTP mixture (0.5 mM), Taq polymerase (0.1 Uµl^−1^), TrisCl (20 mM, PH = 8.3) (Transgen, Beijing, China), as well as 1 µl each primer (5 µM), 1 µl template DNA (c. 30–50 ng genomic DNA), and finally 7 µl distilled deionized water. Polymerase Chain Reactions (PCR) were performed on a Veriti 96 WellThermal Cycler (Applied Biosystems, Foster City, California, USA). The cycling conditions were template denaturation at 97 °C for 3 min followed by 32 cycles of 94 °C for 40 s, 52 °C for 40 s, 72 °C for 1 min, and a final step of 72 °C for 7 min.

PCR products were visualized on 1% TAE agarose gels and then purified using a Sangon Purification Kit (Sangon, Shanghai, China). The purified products were used for bi-directional sequencing using the PCR primers and the PRISM Dye Terminator Cycle Sequencing Ready Reaction Kit (Applied Biosystems, Foster City, CA, USA). The products were run on an ABI 3730 xl DNA Sequencer. All sequences were deposited in GenBank under accession numbers KP213841- KP213854, and KP289037- KP289039 (Table S2).

Eight pairs of nSSR markers with higher genetic information, i.e., Den005, Den007, Den033, Den034, Den036, Den058, Den075 and Den096^43^, were selected to detect the genetic divergence among 18 populations of *Dendrocalamus sinicus*. Loci selected for this study have only one or two alleles among all the genotyped individuals. PCRs for the microsatellites were performed separately for each locus in 20 µl volumes with 8.8 µl ddH_2_O, 0.6 µl of DNA extract, 0.3 µl of each fluorescently labelled primer and 10 µl 2× Taq PCR MasterMix (Transgen, Beijing, China), which comprised of 0.5 mM dNTPs, 100 mM KCl, 3 mM MgCl_2_ and 1 U HotStar Taq. PCR thermal cycling conditions were 3 min denaturation at 94 °C, 35 cycles of 30 s denaturation at 94 °C, 40 s annealing at 48 °C (Den058), 50 °C (Den033 and Den036), 52 °C (Den034, Den075 and Den096) or 61 °C (Den006 and Den007), 50 s extension at 72 °C and a single 7 min extension step at 72 °C. PCR products were scanned with QIAxcel Capillary Gel Electrophoresis (QIAGEN, Irvine, USA). Fragment sizes were determined using QIAxcel BioCaculator and analyzed with the software GenALEx version 6.4^44^.

### Chloroplast DNA analysis

We assembled and edited sequences in Sequencher version 4.1.4 (Gene Codes Corporation, Ann Arbor, Michigan, USA). Multiple alignments of sequences and subsequent manual adjustments were obtained using Geneious Pro version 4.8.5^45^. The inversions were treated as indels according to Simmons & Ochoterena^46^. The matrix of combined cpDNA sequences was constructed for the following analysis since the cpDNA in plants generally do not recombine^47^.

All the combined cpDNA sequences were assigned to different haplotypes using DNASP version 5.10^48^, and relatedness between haplotypes were constructed using TCS program version 1.21^25^ with 95% statistical parsimony criteria. We investigated the distribution of the number of pairwise nucleotide differences for cpDNA sequence data according to the method of Rogers & Harpending^49^.

Phylogenetic relationships between haplotypes of *Dendrocalamus sinicus* were analyzed using Maximum Parsimony (MP) in PAUP* version 4.0 beta 10^50^ with *D. latiflorus* as outgroup. We initially use a Modeltest version 3.7^51^ based on hierarchical Likelihood Ratio Tests (hLRTs) to find a best-fitting model applied to Bayesian Inference analysis. We performed full heuristic tree searches with 1000 random stepwise addition, using the TBR branch swapping, ‘MulTrees’ options. To assess the bootstrap supports, we used 1000 replicates. We also calculated Bayesian posterior probabilities using the software program MrBayes version 3.1.2^52^ with a Monte Carlo Markov Chain (MCMC) length of 2 × 10^6^ generations.

To identify clusters of genetically similar populations, we ran a spatial analysis of molecular variance (SAMOVA) in SAMOVA 1.0^53^ using K = 2–9, and chose the number of groups that gave the highest F_CT_, or the number of groups for which F_CT_ began to plateau^54^. We excluded configurations with one or more single-population groups, because this indicates that the group structure is disappearing^55^. To partition variations within and among defined groups and populations, we performed analysis of molecular variance (AMOVA) using ARLEQUIN version 3.0^56^.

Molecular diversity indices, including the number of haplotypes, haplotype diversity (Hd) and nucleotide diversity (π), were estimated using DNASP version 5.10. The existence of phylogeographic structure was tested following Pons & Petit^57^. Mean genetic diversity within populations (H_S_), total genetic diversity (H_T_), genetic differentiation (G_ST_), and genetic differentiation considered as genetic distance between haplotypes (N_ST_), were obtained using PERMUT version 1.0 (http://www.pierroton.inra.fr/genetics/labo/Software/). A permutation approach was used to test the significance of the differences between G_ST_ and N_ST_^27^ with the same program.

To investigate evidence of recent population expansion, we tested the null hypothesis of spatial expansion using mismatch distribution analysis. The goodness-of-fit was tested with the sum of squared deviations (SSD) between observed and expected mismatch distributions^48^ and Harpending’s raggedness index^58^ (HRag), using a parametric bootstrap approach^59^ with 1000 replicates in Arlequin. We constructed the mismatch distributions of pairwise genetic differences in each group using Dnasp. For the expanding population group identified, we used the formula t = τ/2 u to estimate the time since expansion began. Values for u were calculated as u = μkg, where μ is the number of substitutions per site per year (s/s/y), k is the average sequence length of the cpDNA region under study, and g is the generation time in years. The paleotropical bamboos diversified from an estimated ca. 15 Mya and followed by rapid radiation within the lineages^11^. We adopted the overall synonymous substitution rate of cpDNA in palaeotropical woody bamboos, 9.03 × 10^−10^ s/s/y, as the value of μ^60^. The value for k was 1947 bp, and 76 years was used as an approximation for generation time according to Janzen’s record of *Dendrocalamus giganteus*^61^. We also used Fu’s F_S_^62^ and Tajima’s D^63^ test of selective neutrality to infer demographic history of all populations as well as each group. We estimate the statistical significance by performing 1000 random permutations in Arlequin.

### Microsatellite data analysis

The Hardy-Weinberg equilibrium (HWE) for each population and each locus was tested in FASTAT version 2.9.3.2^64^. We calculated the number of alleles (N_A_), private alleles (N_P_), effective number of alleles (N_E_), expected heterozygosity (H_E_), and observed heterozygosity (H_O_), for each population and group in GenALEx. FSTAT was used to estimate Allelic richness (A_R_), inbreeding index (F_IS_), total genetic diversity (H_T_), genetic differentiation based on the allelic frequencies (G_ST_), and the standardized measure of genetic differentiation (G’_ST_).

To reveal the phylogenetic relationships among populations, we generated a neighbour-joining (NJ) tree with bootstrap values inferred from 1000 replicates using the program PHYLIP version 3.6^65^, based on Nei’s genetic distance^66^ (D) among all pairs of populations estimated in the procedure MSA version 4.05^67^. A further analysis of population genetic admixture was taken in STRUCTURE version 2.3^68^ to conduct a Bayesian analysis at eight SSR loci. For K = 1 to 10, we performed 20 independent runs for 10^5^ iterations after a burn-in period of 10^5^ with no prior information on the origin of individuals. The combination of ‘admixture’ and ‘allele frequencies correlated’ model was used for the analysis. To determine the most probable value of K, we chose the corrected DeltaK method described by Evanno *et al*.^69^, corrected posterior probability method^68^, the MedMeaK and the MaxMeaK methods described by Puechmaille^70^. All the analyses were completed using Kestimator V.1.12^70^. For each K, ΔK was computed based on absolute value of the second-order rate of change of the likelihood distribution. STRUCTURE results were displayed with the software Distruct version 1.1^71^. To gain a clearer view of genetic clusters, we conducted a principal coordinate (PCO) analysis among all 232 individuals in the program GenAlEx^44^.

Analysis of molecular variance (AMOVA) was performed to assess the genetic differentiation among groups and between populations within groups (identified by phylogenetic analyses) using the program Arlequin 3.0^56^. To detect isolation by distance and evaluate the relative influences of gene flow and drift on the population structure at both the species and group levels, we conducted a Mantel test between matrices of pairwise genetic distance (calculated by Arlequin) and geographic distance obtained (calculated by GenAlEx) with 999 random permutations in GenAlEx. We used the BARRIER program version 2.2^72^ to identify geographical locations where major genetic barriers among populations occurred based on 1000 Nei’s genetic distance matrices. The input files for the BARRIER analysis were from two parts— the X/Y coordinates of the original points (locations of the sampled populations) and genetic distance matrix with 100 replications calculated by MSA 4.05.

Finally, to test if the populations had experienced recent historical population declines, we used the program Bottleneck 1.2.02^73^ to examine the deviation from mutation-drift equilibrium. We chose the two-phase model (TPM) and Wilcoxon test based on the type of molecular marker and the number of loci.

### Estimation on migration patterns among the Northern, Southern and intermediate distribution areas of *Dendrocalamus sinicus*

To detect the bidirectional migration rates between different extant distributions of *D. sinicus*, we divided its 18 extant populations into three regions: the Intermediate (including populations 9, 12–14), Northern (main distribution of TZ lineage, including populations 1–8, 10 and 11, named the TZ’ region in Table S4) and Southern (main distribution of WQ lineage, including populations 15–18, named the WQ’ region in Table S4) regions based on distributional geography and haploid composition. The bidirectional migration rates among the three areas were estimated using the program MIGRATE-N 3.6.11^74^. A full model with two migration rates (gene flow in and out of the populations) M = m/μ, where m is the immigration rate per generation among populations and μ is the mutation rate per generation per locus, was evaluated with this program. The model comparison was done using Bayes factors that need the accurate calculation of marginal likelihoods. These likelihoods were calculated using thermodynamic integration in MIGRATE-N 3.6.11. We also used Migrate-N to test the relative support for different migration models to address specific dispersal hypotheses. The probability of four models were evaluated using marginal likelihoods and Bayes factors^74^: (1) a full model with two population sizes and two migration rates (from population A to population B and from B to A), (2) a model with two population sizes and one migration rate to A, (3) a model with two population sizes and one migration rate to B, (4) a model where A and B are part of the same panmictic population. All other settings were at default values except the increment between samples placed at 1,000; samples per replicate was set at 10,000; and burn-ins per replicate were set at 50,000,000, and 1,000 replicates.

The pollen-to-seed migration ratio (r = m_p_/m_s_) was calculated following the formula according to Petit *et al*.^30^.

## Electronic supplementary material

Dataset 1
