# Supplementary material for: Genetic structure and differentiation in Dendrocalamus sinicus (Poaceae: Bambusoideae) populations provide insight into evolutionary history and speciation of woody bamboos
Source: Sci Rep. 2018 Nov 16;8:16933. doi: 10.1038/s41598-018-35269-8 (PMC6240087; doi:10.1038/s41598-018-35269-8)
Supplement: Supplementary file 1 — Dataset 1 [file 41598_2018_35269_MOESM1_ESM.doc]

**Genetic structure and differentiation in *Dendrocalamus sinicus* (Poaceae: Bambusoideae) populations provide insight into evolutionary history and speciation of woody bamboos**

Jun Bo Yang^1，2*^, Yu Ran Dong^1，*^, Khoon Meng Wong^3^, Zhi Jia Gu^1, 2^, Han Qi Yang^1^ and De Zhu Li^2^

**Additional Information**

Dataset: Table S1-S5, Figure S1-S6.

**Supplementary information** accompanies this paper at <http://www.nature.com/srep>

**Table S1** CpDNA sequence polymorphisms detected in three intergenic spacer regions of 11 *Dendrocalamus sinicus* haplotypes.

**Table S2** GenBank accession numbers of all haplotypes detected in the rpl32-trnL, rbcL-psaI and trnG-trnT regions of 232 *Dendrocalamus sinicus* individuals from 18 populations, and three sequences of the outgroup species *D. latiflorus*.

**Table S3** Results of the BOTTLENECK test among 18 populations of *Dendrocalamus sinicus* based on the Wilcoxon test method under a two-phase model (TPM).

**Table S4** Estimation on bidirectional migration rates and gene flow directions among the three regions.

**Table S5** Estimation on ancestral gene flow directions among the three regions.

**Figure S1** Two natural variants of *Dendrocalamus sinicus*, “straight culm” (left) and “sinuous culm” (right).

**Figure S2** Spatial analysis of the molecular variance (SAMOVA) of the 18 *Dendrocalamus sinicus* populations.

**Figure S3** Genetic clustering estimated by STRUCTURE.

**Figure S4** Principal coordinates analysis of 232 individuals from 18 populations with 232 individuals of *Dendrocalamus sinicus* based on SSR data.

**Figure S5** Scatterplots representing relationships between genetic distance (F_ST_) and geographic distance (Km) at species and regional levels based on SSR data, (a) all populations, (b) TZ group, (c) WQ group.

**Figure S6** Results of BARRIER analysis based on Nei’s genetic distance (Nei et al., 1983), uncovering two possible genetic boundaries (red lines) among 18 populations of *Dendrocalamus sinicus* (blue circles), with bootstrap values over 1000 replications indicated.

**Table S1** CpDNA sequence polymorphisms detected in three intergenic spacer regions of 11 *Dendrocalamus sinicus* haplotypes.

| Haplotypes | rpl32-trnL |  | trnG-trnT | | | | | | | |  | rbcL-psaI |
| --- | --- | --- | --- | --- | --- | --- | --- | --- | --- | --- | --- | --- |
|  | 216-221 |  | 754 | 821 | 892 | 1080 | 1109 | 1222 | 1428 | 1438 |  | 1611-1617 |
| H1 | I1 |  | G | T | T | A | A | T | G | G |  | I3 |
| H2 | I2 |  | T | G | C | C | C | G | A | G |  | I4 |
| H3 | I2 |  | G | G | T | A | C | G | A | T |  | I3 |
| H4 | I2 |  | T | G | C | A | A | T | A | G |  | I4 |
| H5 | I2 |  | G | T | T | A | A | T | A | G |  | I4 |
| H6 | I2 |  | T | G | C | C | A | G | G | G |  | I4 |
| H7 | I2 |  | T | G | C | C | A | G | A | G |  | I3 |
| H8 | I2 |  | G | T | T | A | A | T | G | G |  | I3 |
| H9 | I2 |  | T | G | C | C | C | G | G | G |  | I3 |
| H10 | I1 |  | T | G | C | C | A | G | G | G |  | I3 |
| H11 | I1 |  | G | T | T | A | A | G | A | T |  | I3 |

I1: TTTTAT; I2: ATAAAA; I3: TATATTT; I4: AACTATA

**Table S2** GenBank accession numbers of all haplotypes detected in the rpl32-trnL, rbcL-psaI and trnG-trnT regions of 232 *Dendrocalamus sinicus* individuals from 18 populations, and three sequences of the outgroup species *D. latiflorus*.

| Species | Region | Code | GenBank accession number |
| --- | --- | --- | --- |
| *Dendrocalamus latiflorus* | rbcL-psaI | LI | KP289037 |
| *Dendrocalamus latiflorus* | rpl32-trnL | 32L | KP289038 |
| *Dendrocalamus latiflorus* | trnG-trnT | GT | KP289039 |
| *Dendrocalamus sinicus* | rbcL-psaI | LI-1 | KP213841 |
| *Dendrocalamus sinicus* | rbcL-psaI | LI-2 | KP213842 |
| *Dendrocalamus sinicus* | rbcL-psaI | LI-3 | KP213843 |
| *Dendrocalamus sinicus* | rbcL-psaI | LI-4 | KP213844 |
| *Dendrocalamus sinicus* | rbcL-psaI | LI-5 | KP213845 |
| *Dendrocalamus sinicus* | rpl32-trnL | 32L-1 | KP213846 |
| *Dendrocalamus sinicus* | rpl32-trnL | 32L-2 | KP213847 |
| *Dendrocalamus sinicus* | trnG-trnT | GT-1 | KP213848 |
| *Dendrocalamus sinicus* | trnG-trnT | GT-2 | KP213849 |
| *Dendrocalamus sinicus* | trnG-trnT | GT-3 | KP213850 |
| *Dendrocalamus sinicus* | trnG-trnT | GT-4 | KP213851 |
| *Dendrocalamus sinicus* | trnG-trnT | GT-5 | KP213852 |
| *Dendrocalamus sinicus* | trnG-trnT | GT-6 | KP213853 |
| *Dendrocalamus sinicus* | trnG-trnT | GT-7 | KP213854 |

**Table S3** Results of the BOTTLENECK test among 18 populations of *Dendrocalamus sinicus* based on the Wilcoxon test method under a two-phase model (TPM). *, significant (P ≤ 0.05).

| Population code | Sample size | Wilcoxon test P-value  (T.P.M) |
| --- | --- | --- |
| 1 | 12 | 0.01563* |
| 2 | 12 | 0.94531 |
| 3 | 12 | 0.01172* |
| 4 | 14 | 0.02734* |
| 5 | 12 | 0.03906* |
| 6 | 15 | 0.07422 |
| 7 | 13 | 0.01172* |
| 8 | 12 | 0.05469 |
| 9 | 15 | 0.46094 |
| 10 | 15 | 0.05469 |
| 11 | 15 | 0.46094 |
| 12 | 12 | 1.00000 |
| 13 | 12 | 0.64063 |
| 14 | 11 | 0.25000 |
| 15 | 13 | 0.00781* |
| 16 | 12 | 0.64063 |
| 17 | 16 | 0.46094 |
| 18 | 9 | 0.03125* |

**Table S4** Estimation on bidirectional migration rates and gene flow directions among the three regions.

| Data | Parameter | Migration rates |
| --- | --- | --- |
| Three Cp sequences | M _In.→TZ’_ | 0.681 |
|  | M _TZ’→ In_. | 0.617 |
|  | M _In.→WQ’_ | 0.695 |
|  | M _WQ’→In_. | 0.623 |
|  | M _TZ’→WQ’_ | 0.626 |
|  | M _WQ’→TZ’_ | 0.648 |
| SSRs | M _In.→TZ’_ | 0.469 |
|  | M _TZ’→ In_. | 0.372 |
|  | M _In.→WQ’_ | 0.396 |
|  | M _WQ’→In_. | 0.438 |
|  | M _TZ’→WQ’_ | 0.390 |
|  | M _WQ’→TZ’_ | 0.425 |

Table S5 Estimation on ancestral gene flow directions among the three regions.

| Dataset | Populations | model | Bezier ImL | Model probability | Ancestral gene flow direction |
| --- | --- | --- | --- | --- | --- |
| Three Cp sequences | Northern (1, TZ’ ) and the Intermediate (2) | 1 | -2952.73 | 0.002 |  |
|  |  | 2 | -2946.69 | 0.998 | In →TZ’ |
|  |  | 3 | -2966.09 | 0.000 |  |
|  |  | 4 | -2959.19 | 0.000 |  |
|  | the Intermediate (1) and Southern (2, WQ’) | 1 | -2933.38 | 0.010 |  |
|  |  | 2 | -2929.74 | 0.376 |  |
|  |  | 3 | -2929.25 | 0.614 | In →WQ’ |
|  |  | 4 | -2947.13 | 0.000 |  |
| SSRs | Northern (1, TZ’ ) and the Intermediate (2) | 1 | -3454.65 | 1.000 | a full model （M _In.→TZ’_=0.630，M _TZ’→In._=0.599） |
|  |  | 2 | -3509.53 | 0.000 |  |
|  |  | 3 | -3672.46 | 0.000 |  |
|  |  | 4 | -3538.85 | 0.000 |  |
|  | the Intermediate (1) and Southern (2, WQ’) | 1 | -2063.97 | 1.000 | a full model（M _In.→WQ’_=0.714，M  _WQ’→In._=0.691） |
|  |  | 2 | -2104.17 | 0.000 |  |
|  |  | 3 | -2076.47 | 0.000 |  |
|  |  | 4 | -2230.66 | 0.000 |  |

| **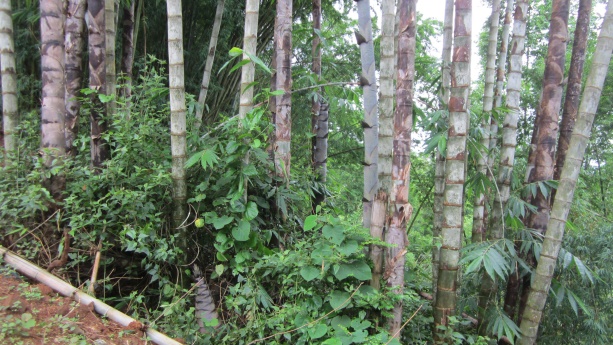** | **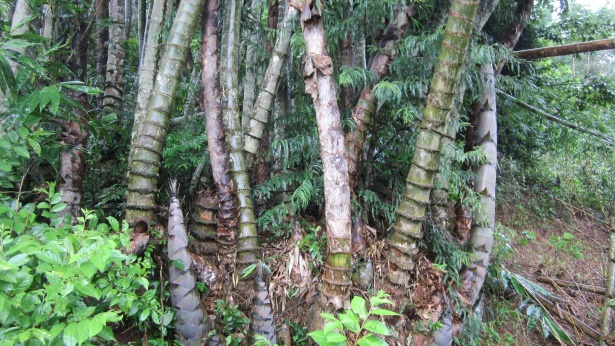** |
| --- | --- |
| straight culm | sinuous culm |

**Fig. S1** Two natural variants of *Dendrocalamus sinicus*, “straight culm” (left, from population ZK) and “sinuous culm” (right, from population JX). Photos by Han-Qi Yang.


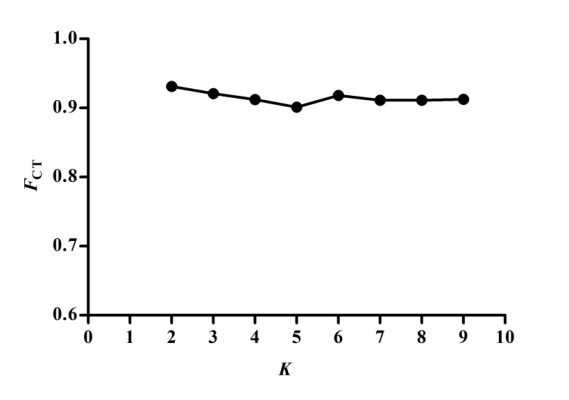


**Fig. S2** Spatial analysis of the molecular variance (SAMOVA) of the 18 *Dendrocalamus sinicus* populations. The percentage variation among groups (F_CT_) is reported for the best-clustering option obtained for each pre-specified value of K (the number of groups), with K ranging from 2 to 9.


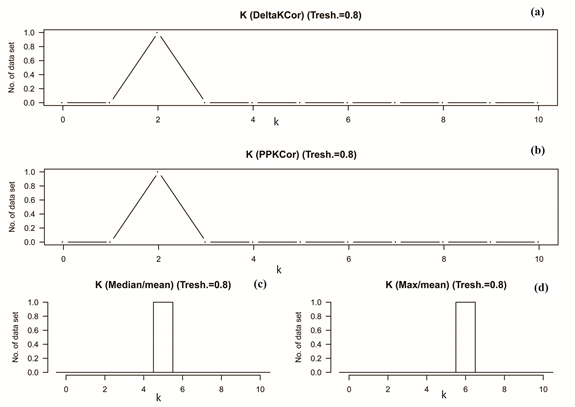


**Fig. S3** Genetic clustering estimated by STRUCTURE. (a) and (b)shows the results of the corrected DeltaK and Posterior probability methods, (c) and (d) shown the results of the ‘MedMeaK' (median of means), the 'MaxMeaK' (maximum of means) methods.


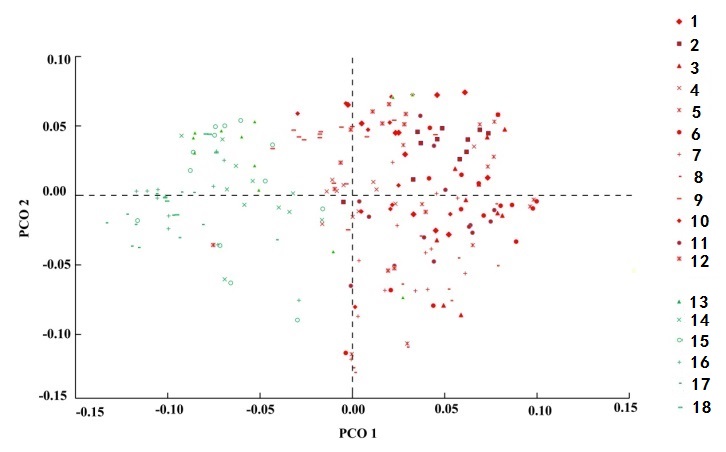


**Fig. S4** Principal coordinates analysis of 232 individuals from 18 populations of *Dendrocalamus sinicus* based on SSR data. The PCO1 and PCO2 axes extracted 28.53% and 18.45% of the total genetic variance, respectively. The numbers 1-18 on the right side of the figure represent population codes, which are identified in Table 1. Color coding corresponds to population clusters (K=2) identified by the STRUCTURE analysis (Fig. 4) and neighbour-joining tree (Fig. 5).


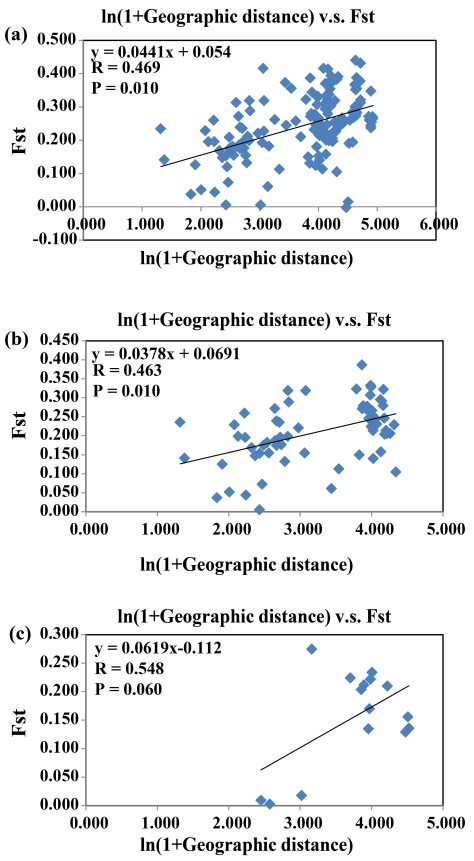


**Fig. S5** Scatterplots representing relationships between genetic distance (F_ST_) and geographic distance (Km) at species and regional levels based on SSR data, (a) all populations, (b) TZ group, (c) WQ group.


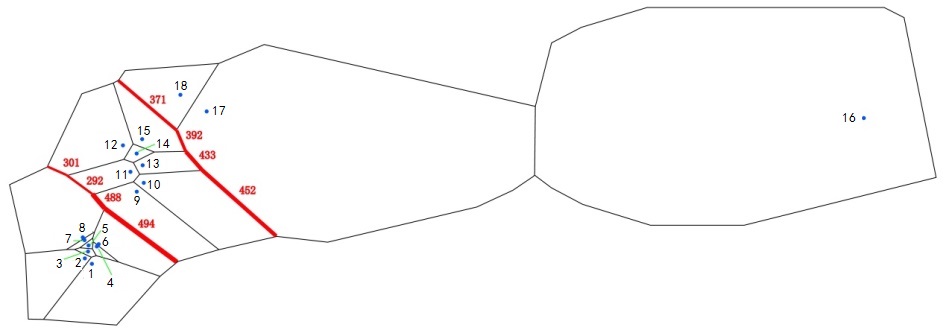


**Fig. S6** Result of BARRIER analysis based on Nei’s genetic distance (Nei et al., 1983), uncovering two possible genetic boundaries (red lines) among 18 populations of *Dendrocalamus sinicus* (blue circles), with bootstrap values over 1000 replications indicated. Black lines represent the Delaunay triangulation.
